# MicroRNA319-TCP19-IAA3.2 Module Mediates Lateral Root Growth in *Populus tomentosa*

**DOI:** 10.3390/plants14162494

**Published:** 2025-08-11

**Authors:** Jianqiu Li, Hanyu Chen, Zhengjie Zhao, Yao Yao, Jiarui Pan, Hong Wang, Di Fan, Keming Luo, Qin Song

**Affiliations:** 1 Key Laboratory of Eco-Environments of Three Gorges Reservoir Region, Ministry of Education, School of Life Sciences, Southwest University, Chongqing 400715, China; 17392708725@163.com (J.L.); gutou289@163.com (H.C.); zzj112022705385278@email.swu.edu.cn (Z.Z.); yaoyao041016@126.com (Y.Y.); pjrokk@126.com (J.P.); wh059260@163.com (H.W.); fandi@swu.edu.cn (D.F.); 2 Key Laboratory of Tree Germplasm Resource Innovation and Utilization, Integrative Science Center of Germplasm Creation in Western China (Chongqing) Science City, School of Life Sciences, Southwest University, Chongqing 400715, China; 3 College of Life Sciences, Northwest A&F University, Xianyang 712100, China

**Keywords:** miR319a, poplar, auxin, TCP19, IAA3.2, lateral root

## Abstract

MicroRNA319 (miR319) and its targets TEOSINTE-BRANCHED1/CYCLOIDEA/PCF (TCP) transcription factors are well-characterized regulators of leaf and flower development, yet their role in root development remains elusive. Here, we demonstrated that overexpression of *miR319a* led to a decrease in the number and density of lateral roots in poplar, while repressing *miR319a* by short tandem target mimics (STTM) promoted lateral root (LR) development. The auxin signaling repressors *IAA3.1* and *IAA3.2* were upregulated in *miR319a*-OE plants but downregulated in *miR319a*-STTM plants. After exogenous applications of naphthaleneacetic acid (NAA), which exhibited the characteristics and physiological functions of the endogenous auxin indole-3-acetic acid, the number and density of LR in WT increased by 30% and 44%, respectively. In *miR319a*-OE plants, the LR number increased by 23% and 48%, and the LR density increased by 10% and 26%. NAA treatment can partially compensate for the phenotype of inhibited LR development caused by the overexpression of *miR319a*. After N-1-naphthylphthalamic acid (NPA) treatment, which is a key inhibitor of the directional (polar) transport of the auxin hormone in plants, the LR number in WT decreased by 70%. In the overexpression plants, the number of lateral roots decreased by 85–87%, and in the STTM plants, the number of lateral roots decreased by about 83%. It was proved that NPA treatment could reverse the phenotype of increased LR number in *miR319a*-STTM plants. Expression analysis revealed that miR319a significantly inhibited the expression of the key auxin-regulated genes *IAA3.1* and *IAA3.2*, suggesting that auxin signaling might mediate its effects on lateral root formation. Additionally, we compared the fluorescence signal in the reporter line with GFP expression driven by the auxin-responsive DR5 promoter within the genetic backgrounds of WT, *miR319a*-OE, and *miR319a*-STTM plants, which revealed that auxin signaling was stronger in the epidermal cells and elongation zone cells in the LR of *miR319a*-OE plants, whereas in LR of WT and *miR319a*-STTM plants, auxin signaling was more pronounced in the root tip meristematic cells. Furthermore, transactivation assays and expression analysis indicated that *IAA3.2* was a downstream target of TCP19. Chromatin immunoprecipitation coupled with quantitative PCR (ChIP-qPCR) confirmed that TCP19 directly bound to the promoter region of *IAA3.2*. These findings establish that miR319a targeted and cleaved *TCP19*, and TCP19 further directly and negatively regulates the expression of *IAA3.2*, thereby controlling LR development in *Populus tomentosa* (*P. tomentosa*). The formation of LR can expand the plant root system, which is of great significance for the vegetative propagation of plants and the in-vitro regeneration of explants. Moreover, the formation of LR is an important strategy for plants to cope with environmental stresses. This study provides a theoretical basis for breeding poplars more suitable for vegetative propagation.

## 1. Introduction

Lateral root morphogenesis constitutes a pivotal process in tree growth and development, with particular significance in vegetative propagation, including cutting propagation [1,2]. Furthermore, lateral roots play an essential role in drought stress adaptation by enhancing hydraulic conductivity and nutrient acquisition efficiency [3]. In poplars, the development of lateral roots has a very important effect on their response to environmental stress. Studies demonstrated that poplars enhance their tolerance to stress such as drought, salt, and low-phosphorus conditions by promoting lateral root formation [4,5,6,7].

Despite its fundamental importance, the molecular regulatory networks and phytohormonal signaling pathways governing lateral root formation in plants remain inadequately characterized. While substantial mechanistic insights have been gained from model herbaceous species, notably *Arabidopsis* [8], the genetic and physiological mechanisms controlling lateral root initiation and elongation in economically important woody species, particularly *Populus*, remain largely unexplored.

Auxin signaling has been unequivocally established as a core regulatory module governing lateral root morphogenesis across diverse plant taxa [8,9]. However, the intricate crosstalk between auxin signaling cascades and other regulatory networks, particularly microRNA-mediated post-transcriptional regulation, remains poorly elucidated. Considering the multifaceted nature of lateral root organogenesis and its critical implications for tree propagation efficiency and stress resilience, comprehensive investigations into the genetic determinants and molecular mechanisms underlying this developmental process are imperative.

MicroRNAs (miRNAs), a class of small non-coding RNAs, emerged as crucial regulators of plant growth, development, and stress responses to both biotic and abiotic stimulus [10]. Among the numerous miRNAs computationally predicted and experimentally validated across plant species [11], the miR319 family represents one of the most evolutionarily ancient and phylogenetically conserved miRNA families [12,13]. MiR319 exerts its regulatory function primarily through post-transcriptional repression of TEOSINTE-BRANCHED1/CYCLOIDEA/PCF (TCP) transcription factors, which are integral to multiple plant developmental pathways [14]. While miR319 has been extensively characterized in leaf morphogenesis and growth regulation [15,16], its role in root development remains underexplored. Previous studies have shown that miR319 participated in the development of reproductive structures, including petals and stamens [17], and contributes to secondary cell wall biosynthesis in stems [18]. In *Populus*, miR319a has been shown to regulate salt tolerance through regulation of xylem vessel morphology, including the vessel number and lumen area, as well as ion transport dynamics [19]. Despite these organ-specific functional characterizations, the precise role of miR319 in root development, particularly in lateral root formation and root system architecture establishment, remains to be elucidated.

Recent studies revealed that miR319 participates in complex cross-talk with multiple phytohormonal signaling pathways to orchestrate plant growth and development. Notably, *TCP19*, a direct target of miR319a, has been demonstrated to physically interact with RGA, a key component of the gibberellin (GA) signaling pathway, thereby regulating trichome initiation [20]. Furthermore, miR319-mediated regulation of *TaPCF8* in wheat has been shown to integrate auxin signaling and biosynthesis pathways, ultimately controlling plant height determination [21]. TCP transcription factors have been increasingly recognized as crucial modulators of auxin signaling dynamics during cellular differentiation and organ maturation processes [22]. Specifically, TCPs influence auxin biosynthesis and response by regulating genes such as *YUC5*, *IAA3*, and *PIN1* [16,23]. Given the central role of auxin signaling in root development [8,24], it remains unclear whether miR319-TCP regulatory networks influence auxin metabolism and signaling to influence lateral root development.

In poplars, current research on the regulation of lateral root development by small RNA-mediated hormone signaling is still limited. In this study, we identified that miR319a/TCP19/IAA3.2 module functioned as a key regulator for LR development in *P. tomentosa*. Genetic modification of *miR319a*/*TCP19* expression and detection of auxin signaling showed the antagonism between the miR319a and auxin pathway in modulating LR growth in *P. tomentosa*. Molecular and biochemical evidence indicate that *TCP19*, as a downstream target of miR319a, directly bound to the promoter of *IAA3.2* and negatively regulated its expression level, thereby altering auxin signaling. These findings establish a novel link between miR319a and lateral root formation, providing new insights into the genetic and molecular regulation of tree growth and propagation.

## 2. Results

### 2.1. MiR319a Modulates Lateral Root Development in P. tomentosa

**Figure 1:**
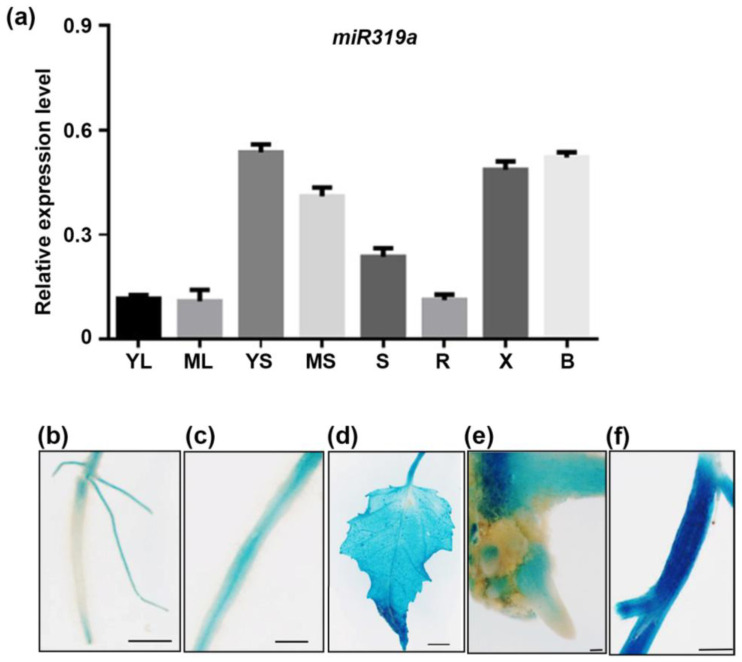
The expression pattern of *miR319a* in poplar. (**a**) The expression pattern of *miR319a*. YL means young leaf; ML means mature leaf; YS means young stem; MS means mature stem; S means shoot; R means root; X means xylem; and B means bark. (**b**–**f**) Histological staining of the root (**b**), lateral root (**c**), leaf (**d**), base of root (**e**), and stem (**f**) in 1-month-old transgenic poplar harboring the GUS reporter gene driven by the promoter of *miR319a*. Bars: (**b**) = 5 mm; (**c**) = 10 mm; (**d**) = 5 mm; (**e**) = 1 mm; and (**f**) = 5 mm; *n* = 3.

The miR319 family in *Populus trichocarpa*, consisting of nine distinct members, was distinguished by a highly conserved 20-nucleotide mature sequence [25]. Previous studies implicated miR319a in diverse biological processes in woody plants [20]. To investigate its expression pattern in *P. tomentosa*, we quantified *miR319a* transcript levels in WT plants across various tissues using stem-loop RT-qPCR. *MiR319a* was ubiquitously expressed, with the highest levels detected in the stem (Figure 1a).

Histochemical staining of *miR319a_pro_:GUS* transgenic plants corroborated the RT-qPCR results, revealing weak GUS activity in the root, lateral root (LR), and root base (Figure 1b,c,e), but stronger signals in the stem and leaf (Figure 1d,f). These findings suggest that miR319a regulated LR development through modulation of auxin source and canalization pathways. The results of tissue expression implicate the possible role of miR319a in LR regulation.

**Figure 2:**
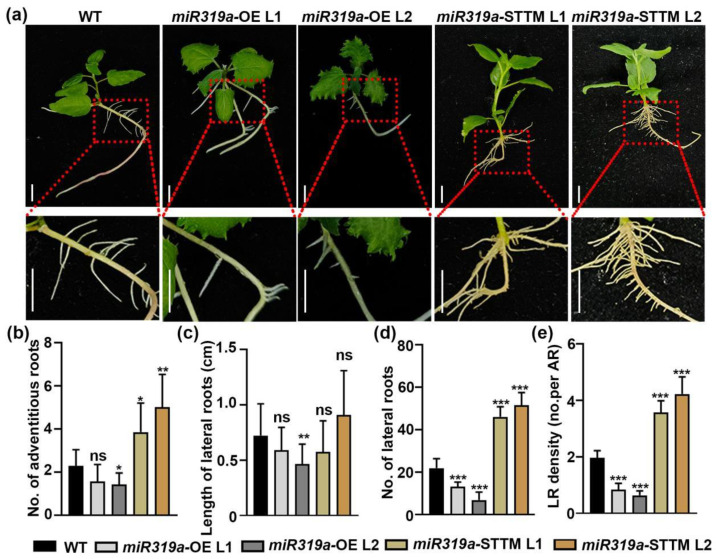
miR319a participates in root development and affects lateral root formation in poplar. (**a**) Hydroponic culturing WT, *miR319a*-OE, and *miR319a*-STTM plants for 15 days to observe the phenotype of roots. The growth conditions of seedling should be similar. Bar (up) = 1 cm; (down) = 3 cm. (**b**) Quantitative measurements of adventitious roots in WT, *miR319a*-STTM, and *miR319a*-OE plants. (**c**) Quantitative measurements of length of lateral roots in WT, *miR319a*-STTM, and *miR319a*-OE plants. (**d**) Quantitative measurements of number of lateral roots in WT, *miR319a*-STTM, and *miR319a*-OE plants. (**e**) Quantitative measurements of density of lateral roots in WT, *miR319a*-STTM, and *miR319a*-OE plants. No. represents number; LR means lateral root, and AR means adventitious root. Student’s *t*-tests were used to analyze the significant statistical differences (* *p* < 0.05, ** *p* < 0.01 and *** *p* < 0.001) and ns means no significant difference; *n* = 5.

We subsequently performed tissue propagation of the *miR319a*-OE and *miR319a*-STTM transgenic *P. tomentosa* plants and analyzed the relative expression level of *miR319a* in root (Figure S1a). Shoots from transgenic plants and WT plants were rooted on woody plant medium (WPM), and root traits were assessed after 14 days (Figure 2a). The number of adventitious roots increased significantly in the *miR319a*-STTM plants, but the difference was not obvious in the overexpression lines (Figure 2b). There were also no significant differences in lateral root length among the WT, *miR319a*-OE, and *miR319a*-STTM transgenic poplars (Figure 2c). However, compared with WT, the number of lateral roots in the *miR319a*-OE L1 and L2 lines decreased by 40% and 69%, respectively (Figure 2d), and the LR density decreased by 57% and 67%, respectively (Figure 2e). In contrast, in *miR319a*-STTM L1 and L2 lines, both the LR number and LR density approximately doubled. Additionally, 10-week-old *miR319a*-OE plants cultivated in soil exhibited higher fresh (Figure S1b,c) and dry root weights (Figure S1b,d) compared to WT plants. These data collectively demonstrate that miR319a acted as a negative regulator of LR development in *P. tomentosa*.

### 2.2. MiR319a Regulates Lateral Root Formation by Modulating Auxin Signaling

**Figure 3:**
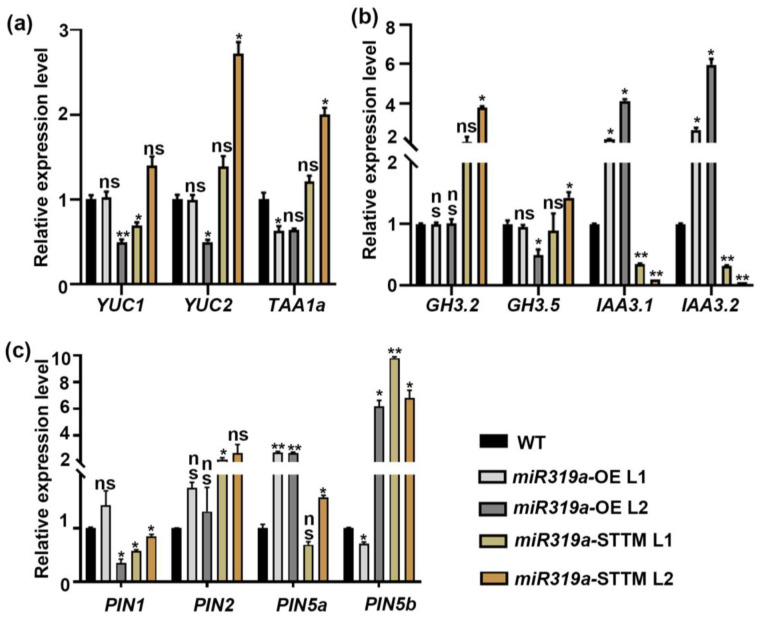
The relative expression level of auxin-related genes in wild-type and miR319a transgenic plants. (**a**–**c**) The relative expression level of the marker genes of auxin biosynthesis (**a**), response (**b**), and transport (**c**). The reference gene was *UBQ*. Student’s *t*-test was used to analyze the significant statistical differences (* *p* < 0.05, ** *p* < 0.01) and ns means no significant difference; *n* = 3.

Auxin has been widely proven to be involved in regulating the development of lateral roots [8,9]. To explore the functional connection between miR319a and auxin signaling during LR development, we analyzed the expression of genes involved in auxin biosynthesis, signaling, and transport in *miR319a*-OE and *miR319a*-STTM plants (Figure 3a–c). For auxin biosynthesis, *YUC2* and *TAA1a* expression levels were reduced in one of the *miR319a*-OE lines and enhanced in one of the *miR319a*-STTM lines (Figure 3a). Further, the auxin signaling repressors *IAA3.1* and *IAA3.2* were upregulated in *miR319a*-OE plants but downregulated in *miR319a*-STTM plants (Figure 3b). The relative expression level of auxin transporters *PIN1*, *PIN2*, *PIN5a, and PIN5b* did not show an obvious expression trend in WT and *miR319a* transgenic plants (Figure 3c). To investigate whether *miR319a* affects the lateral root development by regulating the auxin synthesis or signaling in poplar roots, the auxin content (IBA, MeIAA, and IAA) was detected of WT, *miR319a*-OE, and *miR319a*-STTM roots using liquid chromatography-coupled mass spectrometry (LC-MS) (Figure S2). The results show that compared to WT, in the root of *miR319a*-OE lines, the IBA content was decreased but the IAA content was significantly increased, while both IBA and IAA contents were increased in *miR319a*-STTM plants. Previous studies found that IBA promotes the production of lateral roots in rice more effectively than IAA [26]. In addition, exogenous application of IBA promotes the rooting of poplar tissue-cultured plants [27], while there was no significant difference in the MeIAA content among the WT, *miR319a*-OE, and *miR319a*-STTM plants. Since multiple factors, such as auxin synthesis, transport, and translocation, could affect the changes in auxin content, we speculated that miR319a might influence lateral root formation by modulating auxin levels in the roots.

**Figure 4:**
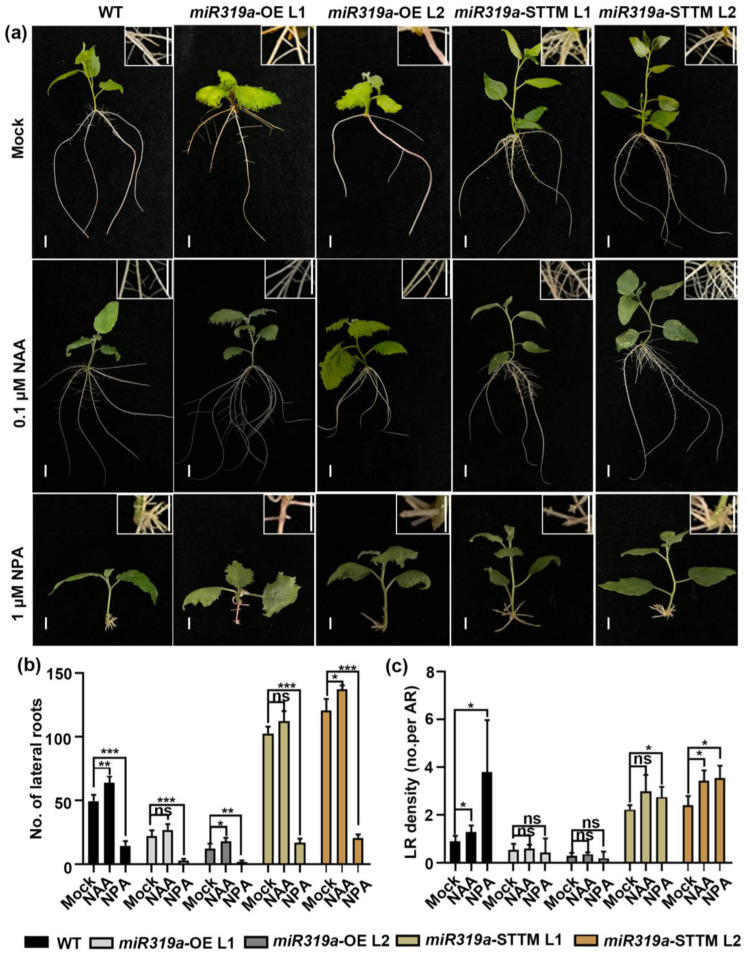
Effects of NAA and NPA on lateral root development upon WT and *miR319a* transgenic plants. (**a**) The WT, *miR319a*-OE, and *miR319a*-STTM plants treated by 0.1 μM NAA and 1 μM NPA for 15 days. Bar = 2 cm. (**b**,**c**) The statistics of lateral root number (**b**) and density (**c**) of wild-type and miR319a transgenic plants. No. means number; LR means lateral root; and AR means adventitious root. Student’s *t*-test was used to analyze the significant statistical differences (* *p* < 0.05; ** *p* < 0.01; and *** *p* < 0.001) and ns means that there is no significant difference; *n* = 5.

To further validate the role of miR319a in auxin signaling, we therefore compromised auxin homeostasis by treating explants with 0.1 µM NAA, a plant growth regulator with auxin-like activity and 1 µM NPA, an inhibitor of polar auxin transport (Figure 4a). After exogenous applications of NAA, the LR number and LR density in WT increased by 30% and 44%, respectively. In the *miR319a*-OE L1 and L2 plants, the LR number increased by 23% and 48%, and the LR density increased by 10% and 26%, respectively. In the *miR319a*-STTM L1 and L2 plants, after NAA treatment, the number of LR increased by 9% and 14%, and the LR density increased by 34% and 42%, respectively. These results indicate that NAA treatment could partially rescue the phenotypes of reduced LR number and density in *miR319a*-OE plants. Conversely, NPA treatment inhibited LR formation across all genotypes, though the inhibitory effect was less pronounced in *miR319a*-STTM plants compared to WT and *miR319a*-OE lines (Figure 4a–c). After NPA treatment, the LR number in WT decreased by 70%. In the *miR319a*-OE plants, the LR number decreased by 85–87%, and in the *miR319a*-STTM plants, the LR number decreased by approximately 83%. This suggested that NPA treatment could reverse the phenotype of increased lateral root number in *miR319a*-STTM plants. Together, these findings demonstrate that miR319a regulated LR formation in *P. tomentosa* by modulating auxin signaling, with *TCP19* likely playing a pivotal role in this process.

**Figure 5:**
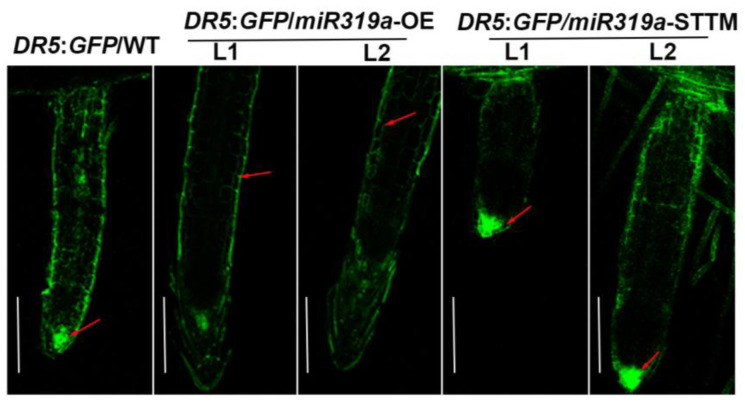
miR319a regulates auxin signaling in lateral root. Detection of GFP fluorescence driven by the auxin-responsive DR5 promoter in LR tips. Bar = 300 μm. The red arrow indicates the location where fluorescence is concentrated. *n* = 3.

IAA3 has been shown to suppress the auxin response [16]. The *IAA3.1* and *IAA3.2* exhibited opposite expression patterns in the *miR319a*-OE lines and *miR319a*-STTM lines, which was consistent with the reduced auxin signaling in the *miR319a*-OE plants (Figure 3b). Furthermore, we transformed the nuclear-localized DR5rev::GFP reporter vector, a biosensor of in vivo auxin signaling, into the genetic backgrounds of WT, *miR319a*-OE, and *miR319a*-STTM, respectively, to investigate the role of miR319a in regulating the auxin pathway during LR formation (Figure 5). The results reveal that auxin signaling was stronger in the epidermal cells and elongation zone cells in the LR of *miR319a*-OE plants, whereas in LR of WT and *miR319a*-STTM plants, auxin signaling was more pronounced in the root tip meristematic cells. This suggested that miR319a might influence lateral root formation by modulating auxin signaling in the root tip meristematic region.

### 2.3. TCP19 Acts as a Target of miR319a

**Figure 6:**
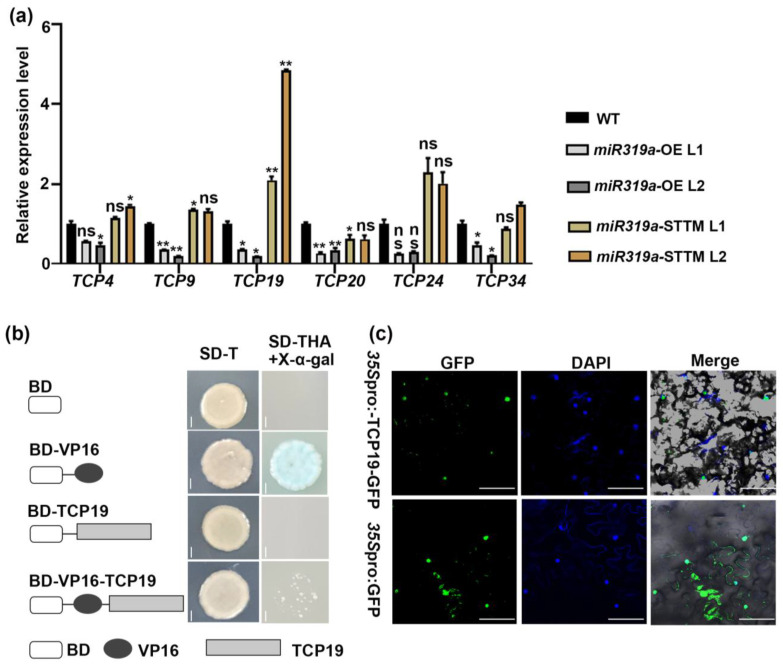
TCP19 is a nucleus-localized activator. (**a**) The relative expression level of TCPs in the root of 2-month-old WT, *miR319a*-OE, and *miR319a*-STTM plants. The reference gene is *UBQ*; *n* = 3 Student’s *t*-tests were used to analyze the significant statistical differences (* *p* < 0.05, ** *p* < 0.01) and ns means that there is no significant difference. (**b**) Transcriptional activity analysis of TCP19. The yeast cells grow on SD medium lacking tryptophan, histidine, and adenine and turn blue after adding X-α-gal. Bar = 0.1 cm. (**c**) Subcellular localization of *35S*pro:TCP19-GFP fusion protein detected via transient expression in tobacco (*Nicotiana benthamiana*) leaf epidermal cells. The nucleus was indicated by DAPI staining. The *35S*pro:GFP vector was used as a control. Bar = 25 μm.

TCP transcription factors were well-established primary targets of miR319, including *TCP2/3/4/10/24* in *Arabidopsis* [15]. In *P. tomentosa*, six TCP genes (*TCP4*, *TCP9*, *TCP19*, *TCP20*, *TCP24*, and *TCP34*) were identified based on sequence homology, all of which were confirmed as direct targets of miR319a [20]. TCP transcription factors have also been increasingly implicated in auxin-related pathways in regulating diverse developmental processes [28,29,30]. To explore the functional connection between miR319a-TCPs and auxin signaling during LR development, we firstly analyzed the expression changes of these TCP genes in the lateral roots of WT and *miR319a* transgenic plants. RT-qPCR analysis demonstrated that the expression of all six *TCP* genes was significantly reduced in the roots of *miR319a*-OE plants, whereas only *TCP19* was markedly upregulated in the roots of *miR319a*-STTM plants. This finding highlights that *TCP19* acted as a key target of miR319a in roots (Figure 6a).

To further explore the role of *TCP19* in LR development, we conducted transcriptional activation assays using the GAL4/VP16 system in yeast cells. Yeast cells expressing either GAL4BD-TCP19 or GAL4BD-VP16-TCP19 recombinant proteins did not grow on selective SD–Trp–His–Ade medium (Figure 6b). In contrast, the GAL4BD-VP16-positive control grew normally and turned blue upon overlay with X-α- gal (Figure 6b), indicating that *TCP19* might function as a transcriptional repressor. We also investigated the subcellular localization of *TCP19* by fusing its full-length CDS to GFP under the control of the *CaMV35S* promoter. The *35S*pro:GFP and *35S*pro:TCP19-GFP constructs were transiently expressed in *Nicotiana benthamiana*. Confocal microscopy showed that *TCP19*-GFP fluorescence was exclusively localized in the nucleus, as indicated by co-localization with nucleus-specific DAPI staining. In contrast, GFP alone exhibited a ubiquitous distribution throughout the cell (Figure 6c). We then performed analysis of histological GUS staining on *GUS* reporter lines driven by the *TCP19* promoter. The results show that it was expressed at relatively high levels in both leaves and lateral roots (Figure S3). These findings preliminarily verify that TCP19 was a nuclear-localized transcriptional repressor, which acted as a target gene of miR319a to regulate LR development.

**Figure 7:**
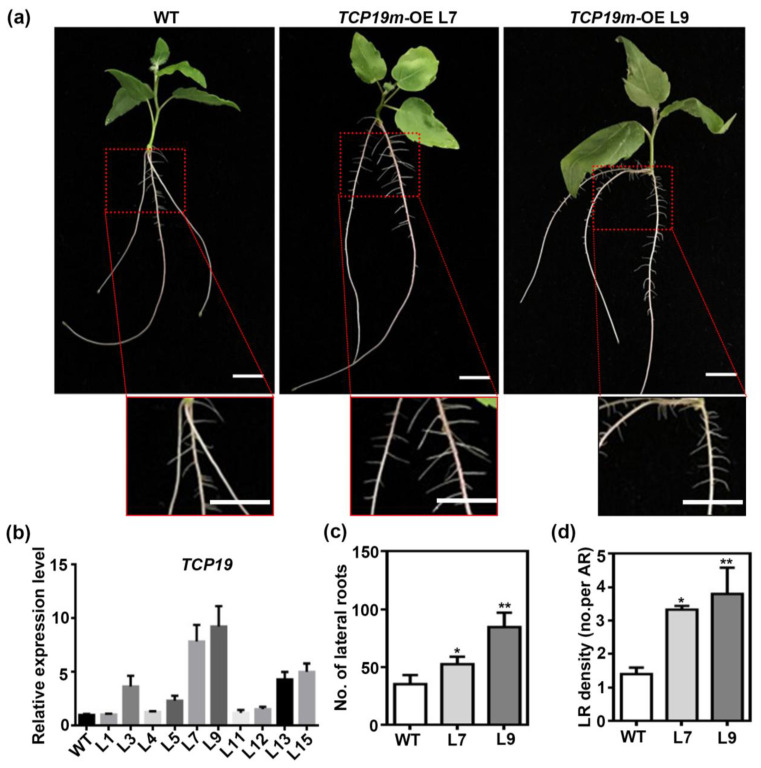
TCP19 positively regulates the lateral root formation. (**a**) Hydroponic culturing wild-type, overexpressing *TCP19m* plants for 15 days to observe the phenotype of roots. Bar = 1 cm. (**b**) The relative expression level of *TCP19* in WT and *TCP19m*-OE poplars. *UBQ* is used as reference gene; *n* = 3. (**c**,**d**) The statistics of LR number (**c**) and LR density (**d**) in WT and *TCP19m*-OE transgenic plants. No represents number; LR means lateral root, and AR means adventitious root. Student’s *t*-test was used to analyze the significant statistical differences (* *p* < 0.05, ** *p* < 0.01); *n* = 5.

To assess the functional role of *TCP19* in LR formation, we overexpressed a mutated version of *TCP19* (*TCP19m*) in *P. tomentosa* under the control of the *CaMV 35S* promoter. Two independent transgenic lines with high expression levels of *TCP19m* (L7 and L9) were selected for further analysis (Figure 7b). Compared to WT plants, *TCP19m-*overexpressing lines displayed a significantly higher number of LR (Figure 7a,c) and increased LR density (Figure 7a,d), confirming that *TCP19* promoted LR growth in poplar.

### 2.4. TCP19 Directly Binds to the Promoter of IAA3.2

Given that TCP19 acts downstream of miR319a in poplar and its overexpression promoted LR development, we hypothesized that TCP19 might regulate auxin-related genes to influence LR development. Previous studies have shown that TCP transcription factors regulate genes involved in auxin biosynthesis, transport, and signaling [16,23,31], and it has been demonstrated that class-I and class-II TCPs preferentially bind to the DNA motifs GGNCCCAC and GTGGNCCC, respectively [32]. Promoter analysis revealed TCP binding elements in the regulatory regions of *IAA3.1* and *IAA3.2*, suggesting potential direct regulation by TCP19 (Figure S4a). Expression pattern analysis further demonstrated that *IAA3.1* is predominantly expressed in young stems (Figure S4b), while *IAA3.2* is mainly expressed in roots (Figure S4c).

**Figure 8:**
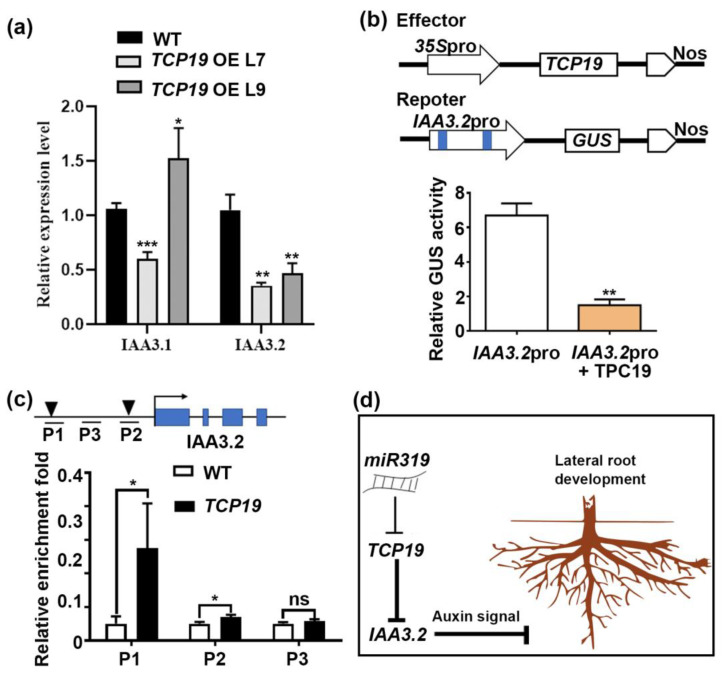
*IAA3.2* is a direct target of TCP19. (**a**) Detection of *IAA3.1* and *IAA3.2* expression in WT and *TCP19m-OE* plants. (**b**) Transient transactivation assays. The effector construct carrying the coding sequence of *TCP19* driven by the *CaMV 35S* promoter. The reporter constructs containing the *GUS* reporter gene were driven by the promoter fragments of *IAA3.2*. The black triangle denotes the position of the binding motif, while the blue box represents the exon region of the gene. (**c**) ChIP-qPCR analysis of the promoter region of *IAA3.2* enriched by TCP19 protein fused with GFP tag. (**d**) Hypothetical model for miR319a/TCP19/IAA3.2-dependent poplar LR growth. Student’s *t*-test was used to analyze the significant statistical differences (* *p* < 0.05; ** *p* < 0.01 and *** *p* < 0.001) and ns means that there is no significant difference; *n* = 3.

To establish whether TCP19 directly regulates *IAA3.2*, we analyzed the expression of *IAA3.1* and *IAA3.2* in the root of *TCP19*m-overexpressing (*TCP19m-OE*) lines. Only *IAA3.2* expression was significantly reduced in these plants (Figure 8a), indicating a TCP19-specific regulation of *IAA3.2*. We further confirmed this interaction using transient expression assays in transiently transformed tobacco leaves. Reporter constructs containing a 2.5-kb promoter fragment of *IAA3.2* driving a *GUS* reporter gene were co-transfected with *TCP19m* effector constructs under the control of the *CaMV 35S* promoter. The result reveals that TCP19 significantly suppressed the promoter activity of *IAA3.2. As* the result shows, in the presence of TCP19, the activity of β-glucuronidase (GUS) decreased by 77%, providing functional evidence of TCP19-dependent repression of IAA3.2 (Figure 8b).

To confirm the direct binding of TCP19 to the *IAA3.2* promoter, we conducted chromatin immunoprecipitation (ChIP) followed by RT-qPCR using *TCP19*m-OE plants. The ChIP enrichment analysis revealed significant binding of TCP19 to the *IAA3.2* promoter regions containing the GGNCCC motif (P1 and P2), in contrast to negative controls (Figure 8c). These results validate that TCP19 directly interacted with the *IAA3.2* promoter, leading to the repression of its expression.

Collectively, these findings establish a regulatory module in which the miR319a-TCP19 pathway suppressed *IAA3.2* expression to regulate auxin-dependent LR development in *P. tomentosa*. This study provided mechanistic insights into the role of miR319a in modulating root architecture through the modulation of auxin signaling in poplar.

## 3. Discussion

MiR319 and its target transcription factors, TCPs, are well-documented regulators of plant development, primarily in aerial tissues. The miR319/TCP module has been implicated in leaf morphogenesis, senescence, and cellular differentiation [33]. In floral development, miR319 mutants exhibit defects in petal and stamen morphology, including impaired anther formation and altered petal size [17]. While significant advances have been made in understanding the role of miR319/TCPs in above-ground organs, their involvement in the regulation of underground tissues, particularly lateral root (LR) development, remains underexplored. Though several studies suggested that miR319 was involved in root responses to various abiotic stresses [34,35], the molecular mechanisms underlying the role of miR319 in LR development have not been fully elucidated. As a herbaceous plant, *Arabidopsis* mainly has fibrous roots. The formation of lateral roots is weakly associated with secondary growth, and auxin relies on short-distance transport in the root tip [8]. In contrast, *P. tomentosa* is a woody plant with a complex root system composed of a taproot and lateral roots. The development of lateral roots in *P. tomentosa* requires coordinated secondary growth. Auxin primarily depends on long-distance transport in vascular tissues, and there are species-specific signal responses [36].

Previous studies on the regulation of lateral roots by miR319/TCP were mostly based on *Arabidopsis*, which made it difficult to reflect the specific mechanisms of woody plants. In this study, we revealed a molecular pathway in which miR319a regulated LR development by targeting TCP19 to inhibit the auxin signaling repressor *IAA3.2* in *P. tomentosa*. These findings fill the gap in the species differences of root regulation mechanisms between herbaceous and woody plants.

### 3.1. Spatial and Temporal Regulation of LR Development by miR319a

MicroRNAs are recognized as key regulators of lateral root development, influencing processes such as initiation, patterning, and emergence. In *Arabidopsis*, miR156 was found to positively regulate lateral root development [37,38]. In poplars, miR390 was demonstrated to mediate salt stress-induced LR formation, enhancing their salt tolerance by promoting root elongation and density. Furthermore, microRNA sequencing in *Citrus* revealed that miR319, in combination with miR171, contributes to root development under long-term boron toxicity [39]. Our analysis of transgenic plants expressing *miR319a*pro:*GUS* confirmed that *miR319a* was expressed at low levels in LR (Figure 1). This temporal expression pattern suggested that miR319a might play a regulatory role in LR growth.

### 3.2. MiR319a Module Modulates LR Development via Auxin Signaling

Previous studies demonstrated that miR319 modulated various hormone signaling pathways, including gibberellin (GA) [20], jasmonic acid (JA) [40], and ethylene (ETH) [35]. Additionally, miR319 has been implicated in regulating auxin signaling in the context of wheat growth [21]. Auxin plays a central role in all stages of LR development, including initiation, patterning, and emergence [41,42,43]. In our study, we observed that overexpression of *miR319a* resulted in a reduction in LR number and density, whereas knockdown of *miR319a* promoted LR formation (Figure 2 and Figure S2). Instead, it might focus more on influencing genes related to auxin synthesis and signal transduction. Notably, among the genes related to auxin transport, the relative expression levels of members of the PIN protein family, such as *PIN1*, *PIN2*, *PIN5a*, and *PIN5b*, did not show obvious regular changes between WT and *miR319a* transgenic plants (Figure 3c). This implied that the regulation of lateral root development by the miR319a/TCP19 module might not be mainly mediated through the regulation of auxin transport by these PIN proteins. Moreover, we found that auxin signaling was enhanced in the roots of *miR319a*-STTM (Figure 3b and Figure 4), suggesting that miR319a negatively regulated LR development by modulating auxin signaling.

### 3.3. Regulation of LR Development via TCP19 and IAA3.2

Although the targets of miR319a include six TCP genes, only *TCP19* was found by us to be significantly up-regulated in *miR319a*-STTM plants, while the expression of other TCP genes showed no obvious changes (Figure 6a). Overexpression of *TCP19* could increase the number and density of LR (Figure 7). These characteristics distinguished it from other TCP genes and made it the core target for miR319a to regulate lateral root development.

TCPs are known to regulate plant development by influencing phytohormone biosynthesis and signaling, particularly auxin [16,35]. In *Arabidopsis*, mutation in *TCP15* results in upregulation of *YUCCA* (*YUC*) genes, which modulates auxin biosynthesis and affects various developmental processes, including gynoecium and silique development [31]. TCP proteins are involved in regulating auxin homeostasis by directly binding to the promoters of auxin-related genes, such as *IAA3/SHY2*, and modulating their expression [44]. Similarly, TCPs modulated auxin homeostasis and respond to affect LR patterning in *Arabidopsis* [45]. In this study, we observed that both *TCP19* and *IAA3.2* were highly expressed in lateral roots, suggesting that they might regulate the LR development in poplars (Figures S3 and S4). Notably, overexpression of *TCP19* increased LR number and density, which correlated with the down regulation of *IAA3.2* (Figure 8a). Motif analysis, transcription activation, and ChIP-qPCR analysis further confirmed that TCP19 directly bound to the promoter of *IAA3.2*, modulating its expression and contributing to LR development (Figure S4a and Figure 8a–c). Interestingly, our work also aligned with Kong et al. [46], who found that TCPs regulate *GH3.3* expression to maintain auxin homeostasis in roots. Additionally, TCPs were demonstrated to coordinate root development in response to environmental cues, such as nutrient availability [31]. Our results extend these findings by providing direct evidence that TCP19 regulates *IAA3.2* expression in poplar, influencing lateral root formation through auxin-mediated pathways.

In summary, we demonstrated that miR319a negatively regulated lateral root development by targeting *TCP19* at the post-transcriptional level. Among the six TCP genes identified, TCP19 appeared to be the primary mediator of miR319a’s effect on lateral root growth. The miR319a/TCP19 module regulated auxin signaling by directly binding to the promoter of *IAA3.2*, influencing lateral root formation in *Populus (*Figure 8d). This work established a novel link between miR319a/TCPs and auxin signaling in lateral root development, providing new insights into the molecular mechanisms controlling root architecture. Future studies should explore the role of other TCP transcription factors and IAA proteins in this regulatory pathway.

## 4. Materials and Methods

### 4.1. Plant Materials, Growth Conditions

All *P*. *tomentosa* (*P. tomentosa*) cutting-propagated plants used in this study were cultivated on woody plant medium (WPM; Coolaber, Beijing, China) in a controlled tissue culture room (Yanghui Instrument, Ningbo, China) under conditions of 16 h of light and 8 h of dark, with 5000 lux supplemental light, 25 °C, and 60% relative humidity.

### 4.2. Poplar Transformation

*Agrobacterium*-mediated transformation was performed using leaf disks as described by Jia et al. [47]. Hygromycin (HYG)-resistant plants were selected using specific HYG primers (Table S1) to identify positive transgenic lines. Ten positive m*TCP19*-OE transgenic plants were screened out via PCR with the gene-specific primers of hygromycin-resistant genes. One-month-old rooted plantlets were transferred to a glasshouse and grown under the same long-day conditions. After one month of cultivation in the greenhouse, the roots of WT and m*TCP19*-OE transgenic plants were used to detect the expression level of *TCP19*.

### 4.3. Gene Cloning and Vector Construction

The miR319a target sites are located within the coding sequence (CDS) of *TCP19*. The *TCP19* gene was amplified from *P. tomentosa* cDNA using gene-specific primers (Table S1) and cloned into the pCXDG vector [48]. Full-length *TCP19* was amplified using specific primer pairs (BD/BD-VP16) and cloned into the BD vector. A 2.5-kb promoter region of *Pro-IAA3.2* was amplified from genomic DNA of *P. tomentosa* and inserted into the pCXGUS-P vector for GUS reporter gene analysis [48], using a TA cloning kit for sequencing. Previous studies generated *TCP19*pro:*GUS, miR319a* pro:*GUS*, *miR319a*-OE, and *miR319a*-STTM-positive transgenic plants, which were selected for this study [20].

### 4.4. Histochemical Analysis

GUS staining was performed as described by Jefferson [49]. Tissues from 15-day-old plants were fixed in 90% acetone for 30 min at 4 °C, then washed three times with double-distilled water (ddH2O). Fixed tissues were incubated in GUS staining solution (0.5 M Tris, pH 7.0, 10% Triton X-100, and 1 mM X-Gluc) for 30 min at 37 °C in the dark. After staining, roots were cleared in 75% ethanol and observed using an Olympus 566 SZX16 microscope (Tokyo, Japan). The GUS staining assays were performed in three biological replicates.

### 4.5. RNA Extraction and Quantitative RT-qPCR

Total RNA was extracted from tissues of root, young stem, mature stem, young leaf, shoot, xylem, and bark in poplars using the Plant RNeasy Mini Kit (Qiagen, Beijing, China). Furthermore, electrophoresis was used to confirm whether the RNA degraded, and the NanoDrop 2000 (Thermo Fisher Scientific, Shanghai, China) was employed to measure the RNA concentration. The DNA in the RNA samples was removed using the DNA eraser in the reverse transcription kit and then reverse-transcribed into cDNA using the PrimeScript™ RT reagent Kit (Takara, Dalian, China) according to the manufacturer’s instructions. A quantitative real-time polymerase chain reaction (qRT-PCR) was performed with SYBR Premix ExTaq™ (Takara) in a qTOWER3G IVD Real-time PCR machine (Analytik Jena AG, Germany). The reaction mixture consisted of 5 μL SYBR Premix ExTaq™, 1 μL cDNA (10 ng), and 0.2 μM of each primer. The *UBQ* gene from *P. tomentosa* was used as a reference gene. Specific primers for qRT-PCR were designed according to Shi et al. [50] (Table S1). The RT-qPCR assays were performed in three biological replicates.

### 4.6. Yeast Transactivation Assay

The full-length open reading frame (ORF) of *TCP19* was amplified using gene-specific primers (Table S1) and cloned into the pGBKT7(BD) and BD-VP16 vector. The pGBKT7 vector and BD-VP16 vector were also transformed into yeast cells, respectively, serving as negative and positive controls. The recombinant plasmids were introduced into the *Saccharomyces cerevisiae* strain Gold2 following the method described by Xu et al. [51]. Yeast transformants were initially grown on SD medium (Coolaber, Beijing, China) lacking tryptophan (Trp) and then further selected on SD medium lacking Trp, histidine (His), and adenine (Ade) for transactivation assays. Transactivation activity was assessed by adding X-α-gal.

### 4.7. Subcellular Localization Analysis

The full-length *TCP19* gene was cloned into the pCXDG vector and fused with GFP to create a *TCP19*-GFP fusion protein. Then, the recombinant plasmid *35S*pro:*TCP19*-GFP was transferred into tobacco leaves using the agroinfiltration method under the condition of *35S*pro:GFP used as a control. The *Nicotiana benthamiana* leaves were stained with 40, 6-diamidino-2-phenylindole (DAPI). Fluorescent signals of GFP and DAPI were documented using a confocal laser microscope (Leica TCS SP8 X; Leica, Wetzlar, Germany).

### 4.8. Morphological Characterization of Roots

For root analysis, 15-day-old WT plants grown on woody plant medium (WPM) were transferred for observation. The number of the adventitious roots and lateral roots of the plants was determined by counting. The poplar plants were photographed, and using the scale used during photography as a parameter, the length of adventitious roots and lateral roots was calculated with the ImageJ software version 1.46r (https://imagej.net/ij, accessed on 1 May 2023) (Bethesda, MD, USA), and the lateral root density of the plant using the known number and length of lateral roots was calculated. Five biological replicates were used for morphological characterization of roots.

### 4.9. Transient Expression and GUS Activity Assay

The full-length *TCP19* fragment was cloned under the control of the 35S CaMV promoter to drive expression as an effector, and the *35S*pro:GFP vector was used as a control. The GUS reporter gene was driven by the *IAA3.2* promoter to serve as the reporter. GUS activity was quantitatively measured by monitoring the cleavage of the β-glucuronidase substrate 4-methylumbelliferyl β-D-glucuronide (MUG), which produces the fluorescent product 4-methylumbelliferone (4MU) upon hydrolysis [49]. The transient expression and GUS activity assays were performed in three biological replicates.

### 4.10. Chromatin Immunoprecipitation Quantitative PCR (ChIP-qPCR)

Two-month-old *TCP19m* transgenic plants containing a GFP epitope were used for ChIP-qPCR assays. Immunoprecipitation was carried out using GFP antibody and normal mouse IgG for negative control. ChIP analysis was performed as described by Yang et al. [52]. The primers used for ChIP-qPCR are listed in Table S1. The ChIP-qPCR assays were performed in three biological replicates.

### 4.11. Determination of Auxin Content by Liquid Chromatography–Mass Spectrometry (LC–MS)

To extract and analyze IAA, IBA, and MeIAA contents in WT and miR319a transgenic plants, the roots were flash-frozen with liquid nitrogen in a mortar right after they were detached from the plants to avoid wounding-induced hormone changes. Then, the frozen tissues were ground into powder. Each sample was accurately weighed at 1.5 g and transferred to 15 mL screw-cap tubes while being kept in liquid nitrogen. In total, 8 μL of 1 μg/mL internal standard working solution was added to each tube. Next, 10 mL of extraction solvent (2-propanol/H_2_O/concentrated HCl at 2:1:0.002, vol/vol/vol) was added, and the volume was adjusted if over 1 g of fresh tissue was used. The tubes were shaken at 100 rpm. for 30 min at 4 °C because plant hormones were unstable. After that, 5 mL dichloromethane was added, and the mixture was shaken in a 4 °C cold room for 30 min. The samples were centrifuged at 4 °C and 13,000× *g* for 5 min. The lower-phase solvent was transferred to a screw-cap vial, concentrated using a nitrogen evaporator, and redissolved in 0.4 mL methanol. The samples were stored at −20 °C until analysis and kept at 8 °C in an autosampler tray during analysis. In total, 2 µL of the sample was injected into a reverse-phase C18 Gemini HPLC column for HPLC–ESI–MS/MS analysis. Finally, quantitative analysis was performed by calculating the ‘correction factor’ of each plant hormone relative to its internal standard. The hormone amounts were normalized to the mass of fresh tissue, and the Analyst 1.5 software was used if an Applied Biosystems triple quadrupole MS was applied.

### 4.12. Statistical Analyses

Quantitative data for expression level analyses, GUS activity measurements, fluorescence intensity measurements, and phenotype data measurements were determined for statistical significance using Student’s *t*-test performed to distinguish significant differences between pairwise samples (*, *p* < 0.05; **, *p* < 0.01; and ***, and *p* < 0.001).

### 4.13. Accession Numbers

The sequences used in this study are available in Phytozome (version 11.0, https://phytozome-next.jgi.doe.gov/blast-search, accessed on 1 May 2023) under the following accession numbers: *TCP19* (Potri.011G083100.4), *TCP9* (Potri.004G065800.5), *TCP4* (Potri.001G375800.1), *TCP20* (Potri.011G096600.2), *TCP24* (Potri.013G119400.1), *TCP34* (Potri.019G091300.1), miR319a (PRJNA611665), *IAA3.1* (Potri.005G053800.1), *IAA3.2* (Potri.013G041300.1), *GH3.2* (Potri.001G298300.1), *GH3.5* (Potri.001G410400.1), *PIN1a* (Potri.012G047200.1), *PIN2* (Potri.018G139400.1), *PIN5a* (Potri.004G124200.1), *PIN5b* (Potri.017G078300.1), *YUC1* (Potri.006G248200), and *TAA1a* (Potri.012G083300).

## 5. Conclusions

In this study, we identified that the miR319a/TCP19/IAA3.2 module regulated lateral root development in poplar by altering auxin signaling. The detection of the expression levels of auxin-related genes and the observation of DR5rev::GFP fluorescent reporter lines demonstrated that miR319a inhibited auxin signaling in lateral roots, thereby suppressing LR development. Further investigation revealed that TCP19, a transcription repressor localized in the nucleus, was the target gene through which miR319a regulates lateral root development. Overexpression of TCP19 could promote lateral root development in poplar. In addition, TCP19 suppressed the expression of the auxin repressor *IAA3.2* by directly binding to its promoter, altering auxin signaling and consequently regulating lateral root development in poplar.

## Data Availability

The original contributions presented in this study are included in the article/Supplementary Materials. Further inquiries can be directed to the corresponding author. The sequences used in this study are available in Phytozome (version 11.0, https://phytozome-next.jgi.doe.gov/blast-search, accessed on 1 May 2023).

## Supplementary Materials

The following supporting information can be downloaded at: https://www.mdpi.com/article/10.3390/plants14162494/s1, Figure S1: miR319a negatively regulates plant growth in poplar. Figure S2: Measurement of endogenous auxin content. Figure S3: The expression pattern of TCP19 in poplar. Figure S4: Promoter analyze of auxin related genes which was regulated. Table S1: Sequences of oligonucleotide primers and probes used in this study.

## References

[1] Preece J.E. (2003). A century of progress with vegetative plant propagation. HortScience.

[2] Miloradovic van Doorn M., Merl-Pham J., Ghirardo A., Fink S., Polle A., Schnitzler J.P., Rosenkranz M. (2020). Root isoprene formation alters lateral root development. Plant Cell Environ..

[3] Bao Y., Aggarwal P., Robbins N.E., Sturrock C.J., Thompson M.C., Tan H.Q., Tham C., Duan L., Rodriguez P.L., Vernoux T. (2014). Plant roots use a patterning mechanism to position lateral root branches toward available water. Proc. Natl. Acad. Sci. USA.

[4] Dash M., Yordanov Y.S., Georgieva T., Tschaplinski T.J., Yordanova E., Busov V. (2017). Poplar PtabZIP1-like enhances lateral root formation and biomass growth under drought stress. Plant J..

[5] Liu S.J., Zhang H., Jin X.-T., Niu M.X., Feng C.H., Liu X., Liu C., Wang H.-L., Yin W., Xia X. (2025). PeFUS3 drives lateral root growth via auxin and aba signalling under drought stress in Populus. Plant Cell Environ..

[6] Fang Q., Jiang T., Xu L., Liu H., Mao H., Wang X., Jiao B.O., Duan Y., Wang Q., Dong Q. (2017). A salt-stress-regulator from the Poplar R2R3 MYB family integrates the regulation of lateral root emergence and ABA signaling to mediate salt stress tolerance in *Arabidopsis*. Plant Physiol. Biochem..

[7] Dash M., Yordanov Y.S., Georgieva T., Kumari S., Wei H., Busov V. (2016). A network of genes associated with poplar root development in response to low nitrogen. Plant Signal. Behav..

[8] Lavenus J., Goh T., Roberts I., Guyomarc’h S., Lucas M., De Smet I., Fukaki H., Beeckman T., Bennett M., Laplaze L. (2013). Lateral root development in *Arabidopsis*, fifty shades of auxin. Trends Plant Sci..

[9] Morffy N.J., Strader L.C. (2018). Locally Sourced, auxin biosynthesis and transport in the root meristem. Dev. Cell.

[10] Chen X. (2008). MicroRNA metabolism in plants. Curr. Top. Microbiol. Immunol..

[11] Taylor R.S., Tarver J.E.B., Hiscock S.J., Donoghue P.C. (2014). Evolutionary history of plant microRNAs. Trends Plant Sci..

[12] Cuperus J.T., Fahlgren N., Carrington J.C. (2011). Evolution and functional diversification of MIRNA genes. Plant Cell.

[13] Yin Z., Li Y., Zhu W., Fu X., Han X., Wang J., Lin H., Ye W. (2018). Identification, characterization, and expression patterns of TCP genes and microRNA319 in cotton. Int. J. Mol. Sci..

[14] Palatnik J.F., Wollmann H., Schommer C., Schwab R., Boisbouvier J., Rodriguez R., Warthmann N., Allen E., Dezulian T., Huson D. (2007). Sequence and expression differences underlie functional specialization of *Arabidopsis* microRNAs miR159 and miR319. Dev. Cell.

[15] Palatnik J.F., Allen E., Wu X., Schommer C., Schwab R., Carrington J.C., Weigel D. (2003). Control of leaf morphogenesis by microRNAs. Nature.

[16] Koyama T., Mitsuda N., Seki M., Shinozaki K., Ohme-Takagi M. (2010). TCP transcription factors regulate the activities of ASYMMETRIC LEAVES1 and miR164, as well as the auxin response, during differentiation of leaves in *Arabidopsis*. Plant Cell.

[17] Nag A., King S., Jack T. (2009). miR319a targeting of TCP4 is critical for petal growth and development in *Arabidopsis*. Proc. Natl. Acad. Sci. USA.

[18] Sun X., Wang C., Xiang N., Li X., Yang S., Du J., Yang Y., Yang Y. (2017). Activation of secondary cell wall biosynthesis by miR319-targeted TCP4 transcription factor. Plant Biotechnol. J..

[19] Cheng Y., Wang Q., Yang L., Li Q., Yan X. (2024). MiR319a-mediated salt stress response in poplar. Hortic. Res..

[20] Fan D., Ran L., Hu J., Ye X., Xu D., Li J., Su H., Wang X., Ren S., Luo K. (2020). The miR319a/TCP module and DELLA protein regulate synergistically trichome initiation and improve insect defenses in *Populus tomentosa*. New Phytol..

[21] Hao P., Jian C., Hao C., Liu S., Hou J., Liu H., Liu H., Zhang X., Zhao H., Li T. (2024). Coordination of miR319-TaPCF8 with TaSPL14 orchestrates auxin signaling and biosynthesis to regulate plant height in common wheat. J. Integr. Plant Biol..

[22] Nicolas M., Cubas P. (2016). TCP factors, new kids on the signaling block. Curr. Opin. Plant Biol..

[23] Challa K.R., Aggarwal P., Nath U. (2016). Activation of YUCCA5 by the transcription factor tcp4 integrates developmental and environmental signals to promote hypocotyl elongation in *Arabidopsis*. Plant Cell.

[24] Yun F., Liu H., Deng Y., Hou X., Liao W. (2023). The role of light-regulated auxin signaling in root development. Int. J. Mol. Sci..

[25] Tuskan G.A., Difazio S., Jansson S., Bohlmann J., Grigoriev I., Hellsten U., Putnam N., Ralph S., Rombauts S., Salamov A. (2006). The genome of black cottonwood, *Populus trichocarpa* (Torr. & Gray). Science.

[26] Wang S., Taketa S., Ichii M., Xu L., Xia K., Zhou X. (2003). Lateral root formation in rice (*Oryza sativa* L.): Differential effects of indole-3-acetic acid and indole-3-butyric acid. Plant Growth Regul..

[27] Lambardi M., Caccavale A.F. (2000). Cryopreservation of white poplar (*Populus alba* L.) by vitrification of in vitro-grown shoot tips. Plant Cell Rep..

[28] Baulies J.L., Bresso E.G., Goldy C., Palatnik J.F., Schommer C. (2022). Potent inhibition of TCP transcription factors by miR319 ensures proper root growth in *Arabidopsis*. Plant Mol. Biol..

[29] Nicolas M., Torres-Perez R., Wahl V., Cruz-Oró E., Rodríguez-Buey M.L., Zamarreño A.M., Martín-Jouve B., García-Mina J.M., Oliveros J.C., Prat S. (2022). Spatial control of potato tuberization by the TCP transcription factor BRANCHED1b. Nat. Plants.

[30] Zhou Y., Zhang D., An J., Yin H., Fang S., Chu J., Zhao Y., Li J. (2018). TCP Transcription factors regulate shade avoidance via directly mediating the expression of both phytochrome interacting factors and auxin biosynthetic genes. Plant Physiol..

[31] Lucero L.E., Uberti-Manassero N.G., Arce A.L., Colombatti F., Alemano S.G., Gonzalez D.H. (2015). TCP15 modulates cytokinin and auxin responses during gynoecium development in *Arabidopsis*. Plant J..

[32] Kosugi S., Ohashi Y. (2002). DNA binding and dimerization specificity and potential targets for the TCP protein family. Plant J..

[33] Schommer C., Bresso E.G., Spinelli S.V., Palatnik J.F., Sunkar R. (2012). Role of MicroRNA miR319 in Plant Development. MicroRNAs in Plant Development and Stress Responses.

[34] Thiebaut F., Rojas C.A., Almeida K.L., Grativol C., Domiciano G.C., Lamb C.R.C., de Almeida Engler J., Hemerly A.S., Ferreira P.C. (2012). Regulation of miR319 during cold stress in sugarcane. Plant Cell Environ..

[35] Liu Y., Li D., Yan J., Wang K., Luo H., Zhang W. (2019). MiR319 mediated salt tolerance by ethylene. Plant Biotechnol. J..

[36] Xu C., Tao Y., Fu X., Guo L., Xing H., Li C., Yang Z., Su H., Wang X., Hu J. (2021). The microRNA476a-RFL module regulates adventitious root formation through a mitochondria-dependent pathway in Populus. New Phytol..

[37] Yu N., Niu Q.W., Ng K.H., Chua N.H. (2015). The role of miR156/SPLs modules in *Arabidopsis* lateral root development. Plant J..

[38] He F., Xu C., Fu X., Shen Y., Guo L., Leng M., Luo K. (2018). The MicroRNA390/trans-acting short interfering RNA3 module mediates lateral root growth under salt stress via the auxin pathway. Plant Physiol..

[39] Huang J.H., Lin X.J., Zhang L.Y., Wang X.D., Fan G.C., Chen L.S. (2019). MicroRNA sequencing revealed citrus adaptation to long-term boron toxicity through modulation of root development by miR319 and miR171. Int. J. Mol. Sci..

[40] Schommer C., Palatnik J.F., Aggarwal P., Chételat A., Cubas P., Farmer E.E., Nath U., Weigel D. (2008). Control of jasmonate biosynthesis and senescence by miR319 targets. PLoS Biol..

[41] De Smet I., Tetsumura T., De Rybel B., Frey N.F.D., Laplaze L., Casimiro I., Swarup R., Naudts M., Vanneste S., Audenaert D. (2007). Auxin-dependent regulation of lateral root positioning in the basal meristem of *Arabidopsis*. Development.

[42] Péret B., De Rybel B., Casimiro I., Benková E., Swarup R., Laplaze L., Beeckman T., Bennett M.J. (2009). *Arabidopsis* lateral root development, an emerging story. Trends Plant Sci..

[43] Tian H., De Smet I., Ding Z. (2014). Shaping a root system, regulating lateral versus primary root growth. Trends Plant Sci..

[44] Tian Q., Uhlir N.J., Reed J.W. (2002). *Arabidopsis* SHY2/IAA3 inhibits auxin-regulated gene expression. Plant Cell.

[45] Das Gupta M., Aggarwal P., Nath U. (2014). CINCINNATA in Antirrhinum majus directly modulates genes involved in cytokinin and auxin signaling. New Phytol..

[46] Kong Q., Low P.M., Lim A.R., Yang Y., Yuan L., Ma W. (2022). Functional antagonism of WRI1 and TCP20 modulates GH3.3 expression to maintain auxin homeostasis in roots. Plants.

[47] Jia Z., Sun Y., Yuan L., Tian Q., Luo K. (2010). The Chitinase Gene (Bbchit1) from *Beauveria Bassiana* enhances resistance to *Cytospora Chrysosperma* in *Populus tomentosa* Carr. Biotechnol. Lett..

[48] Chen S., Songkumarn P., Liu J., Wang G.L. (2009). A versatile zero background T-vector system for gene cloning and functional genomics. Plant Physiol..

[49] Jefferson R.A. (1987). Assaying chimeric genes in plants, the GUS gene fusion system. Plant Mol. Biol. Rep..

[50] Shi R., Sun Y.H., Li Q., Heber S., Sederoff R., Chiang V.L. (2010). Towards a systems approach for lignin biosynthesis in *Populus trichocarpa*: Transcript abundance and specificity of the monolignol biosynthetic genes. Plant Cell Physiol..

[51] Xu C., Fu X., Liu R., Guo L., Ran L., Li C., Tian Q., Jiao B., Wang B., Luo K. (2017). PtoMYB170 positively regulates lignin deposition during wood formation in poplar and confers drought tolerance in transgenic *Arabidopsis*. Tree Physiol..

[52] Yang H., Han Z., Cao Y., Fan D., Li H., Mo H., Feng Y., Liu L., Wang Z., Yue Y. (2012). A companion cell-dominant and developmentally regulated H3K4 demethylase controls flowering time in *Arabidopsis* via the repression of FLC expression. PLoS Genet..

