# Supplementary material for: MicroRNA319-TCP19-IAA3.2 Module Mediates Lateral Root Growth in Populus tomentosa
Source: Plants (Basel). 2025 Aug 11;14(16):2494. doi: 10.3390/plants14162494 (PMC12388896; doi:10.3390/plants14162494)
Supplement: Supplementary file 1 [file plants-14-02494-s001.zip › Supplemental data-clean (20250811).pdf]

SUPPLEMENTAL FIGURES

Figure S1

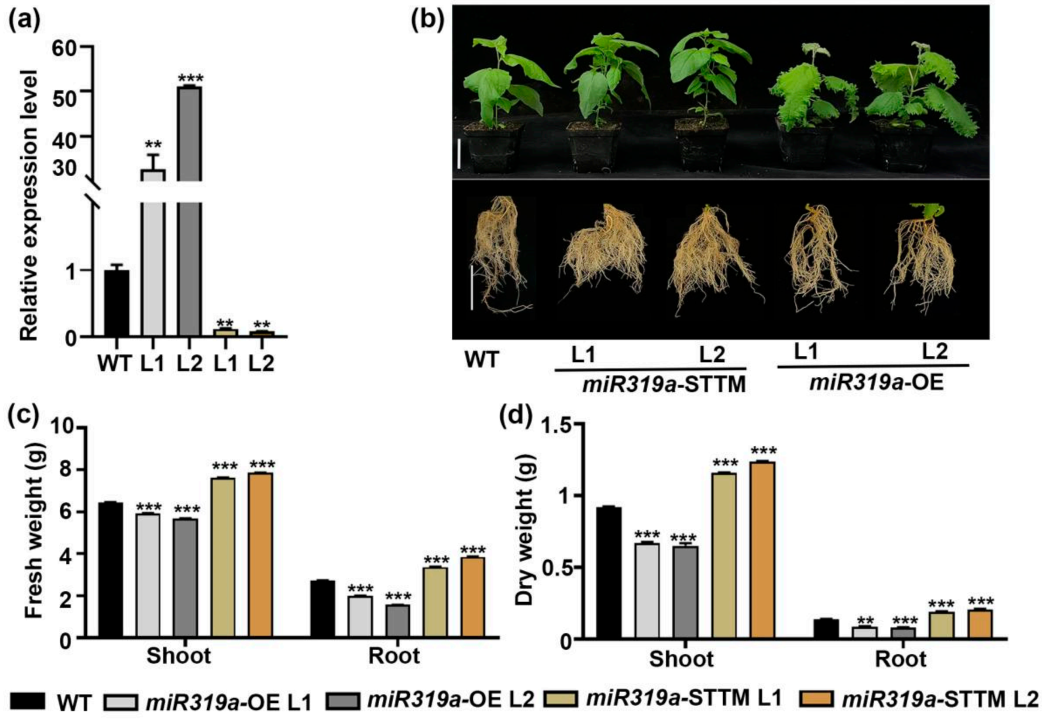

Figure S1. *miR319a* negatively regulates plant growth in poplar.

(a) The expression pattern of *miR319a* in WT, *miR319a* -OE and *miR319a* -STTM lines. (b) Shoot and root phenotypes of wild-type (WT), *miR319a* -STTM, and *miR319a* -OE plants cultivated in soil for 2 months. Bars = 5 cm. (c-d) Fresh weight (c) and dry weight(d) of shoot and root biomass in WT, *miR319a* -STTM, and *miR319a* -OE plants cultivated in soil for 2 months. Student's *t*-tests was used to analyze the significant statistical differences (***P*<0.01, *** *P*<0.001) and ns means that there's no significant difference; *n*=3.

Figure S2

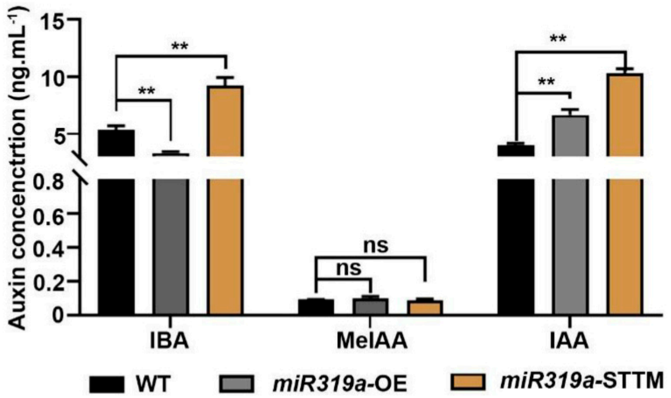

Figure S2. Measurement of endogenous auxin content

Measurement of IBA, MeIAA and IAA in the root of 2-month-old WT, *miR319a* -OE and *miR319a* -STTM plants. Student's *t*-tests was used to analyze the significant statistical differences (**P<0.01) and ns means that there's no significant difference; n=3.

Figure S3

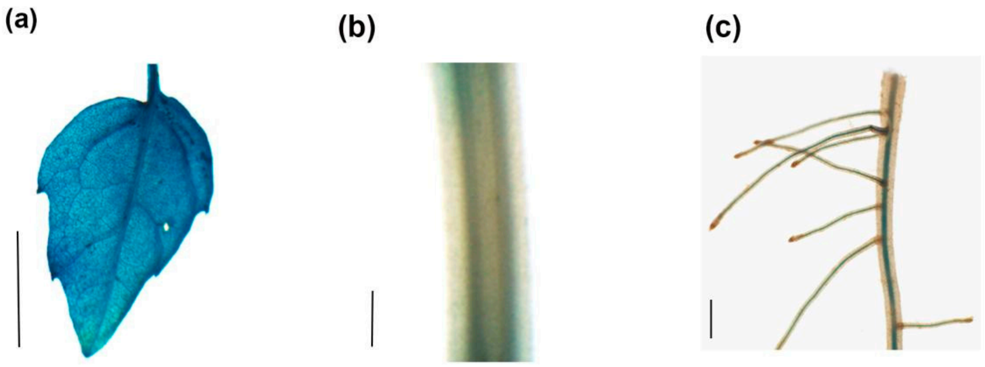

Figure S3. The expression pattern of *TCP19* in poplar

(a-c) Histological staining of the leaf (a), stem(b) and root(c) in 1-month-old transgenic poplar harboring the GUS reporter gene driven by the promoter of *TCP19*. Bars: a=0.5 cm; b=5 mm; c=5 mm.

Figure S4

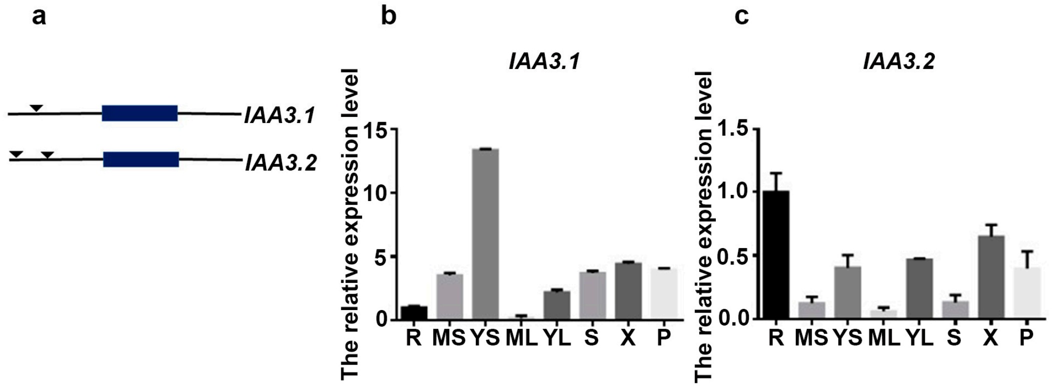

Figure S4. Promoter analyze of auxin related genes which was regulated

(a) Binding sites analysis of TCPs transcription factor. ▼ Represents binding site; blue box represents transcript sequence; black solid line represents non-coding sequence. (b) The expression pattern of *IAA3.1* and *IAA3.2*. R means root; MS means mature stem; YS means young stem; ML means mature leaf; ML means mature leaf; YL means young leaf; S means shoot; X means xylem; P means phloem; *n*=3.
