# Supplementary material for: MicroRNA319-TCP19-IAA3.2 Module Mediates Lateral Root Growth in Populus tomentosa
Source: Plants (Basel). 2025 Aug 11;14(16):2494. doi: 10.3390/plants14162494 (PMC12388896; doi:10.3390/plants14162494)
Supplement: Supplementary file 1 [file plants-14-02494-s001.zip › Table S1.pdf]

Table S1. Sequences of oligonucleotide primers and probes used in this study

Name	Sequence
**Primer for gene cloning and plasmid construction**	
TCP19--fw	5’ TATGGAGGCGGAAGAGATTCG 3’
TCP19-rv	5’ AAGGCAAAGGGAAGAACTGA 3’
mTCP19--fw	5’ AGGGGGACCTTGCAAAGTAAC 3’
mTCP19-rv	5’ GTTACTTTGCAAGGTCCCCCT 3’
IAA3.2-CDS-XhoI-fw	5’ CCGCTCGAGATGGAATTTGAGAGAGATC 3’
IAA3.2-CDS-Spe I-rv	5’ CGGACTAGTCTTTACACAGCACAACCCA 3’
Pro-miR319a-fw	5’ CGTCCATAGACCACAA 3’
Pro- miR319a-rv	5’ AAATGCGACAAACAAATTG 3’
Pro-IAA3.2-fw	5’ TGTCCAGGTCAGCTTGCGCGCAT 3’
Pro-IAA3.2-rv	5’ GATCTCTCTCAAATTCCATACT 3’
**Primer for yeast experiment**	
BD-TCP19-fw	5’CCGGAATTCATGGAGGCGGAAGAGATTC 3’
BD-TCP19-rv	5’ACGCGTCGACTCAGTTCTTCCCTTTGCCTTTC 3’
BD(VP16)-TCP19-fw	5’ TCAGAGGAGGACCTGCATATGATGGAGGCGGAAGAGATTC 3’
BD(VP16)-TCP19-rv	5’ ATCGGTCGGGGGGGCGAATTCCGACTCAGTTCTTCCCTTTGCCTTTC 3’
**Primer for chromatin immunoprecipitation (ChIP) assay**	
ProIAA3.2(CHIP-P1)-fw	5’TGGAAGGAGAGTTAGGGGCA3’
ProIAA3.2(CHIP-P1)-rv	5’AGACTCAGTGGATAGGTAAC 3’
ProIAA3.2(CHIP-P2)-fw	5’TAAGCCCTAGGGTCTCCC 3’
ProIAA3.2(CHIP-P2)-rv	5’GGGGACACAGCTGAGCCG 3’
ProIAA3.2(CHIP-P3)-fw	5’TAAGAATTATTGATGCTTATC 3’
ProIAA3.2(CHIP-P3)-rv	5’GCCATAGGAAGAAGCTATGG 3’
**Primer for PCR genotyping**	
Hyg-fw	5’ CTTCTACACAGCCATCGGTCCAGA 3’
Hyg-rv	5’ GATGTAGGAGGGCGTGGATATGTC 3’
Kana-fw	5’ GTCGACATGGATGGATTGCACG 3’
Kana-rv	5’ GTCGACTCAGAAGAACTCGTCAAGAAG 3’
**Primer for qRT-PCR**	
TCP19-q-fw	5’ TTAGTCGTGATAAGGCTCG 3’
TCP19-q-rv	5’ TGTCATTGCTTGTGGGAT 3’
TCP9-q-fw	5’ GAACTCAATCCCACAACC 3’
TCP9-q-rv	5’ TTCCATCGCCATAACAGA 3’

TCP4-q-fw	5’ GCAGCTGGCGGCGGCG 3’
TCP4-q-rv	5’ GAACTCACCACCTGAGGC 3’
TCP20-q-fw	5’ TGGCGGTGGAGGAAGT 3’
TCP20-q-rv	5’ AGGAAGCAGAGGACGG 3’
TCP24-q-fw	5’ GCTTGGAGCAACGACA 3’
TCP24-q-rv	5’ TTTGGAGAAGAGGGTG 3’
TCP34-q-fw	5’ CCACCTTTGACACCAC 3'
TCP34-q-rv	5’ ACATTTTGCTCATCCC 3’
IAA3.2-q-fw	5’ TGGTGCTCCTTATCTCA 3’
IAA3.2-q-rv	5’ ACAGCACAACCCAAAC 3’
IAA3.1-q-fw	5’ GCTGCTGGACTATACGT 3’
IAA3.1-q-rv	5’ TAGCCTCCGACTCTTT 3’
PtoPIN1a-q-fw	5’ACCAGCAGTTCAAGAACCAG 3’
PtoPIN1a-q-rv	5’AAAGTGGCACCATTGCAGTC 3’
PtoPIN2-q-fw	5’GGGTGCAAGTTTCCTCAAAA 3’
PtoPIN2-q-rv	5’GGCATCTTGATGTTCCACCT 3’
PtoPIN5a-q-fw	5’CGAAGGTTCAGACGTAGCTAAATATT 3’
PtoPIN5a-q-rv	5’AACAAACAAAGCGGTTTATGG 3’
PtoPIN5b-q-fw	5’TCCTTTCCAAGGTGCTCACT 3’
PtoPIN5b-rv	5’AACAAACAAAGCGGTTTATAG 3’
GH3.2-q-fw	5’ AGCTGGATCGTCGCCAATTACTG 3’
GH3.2-q-rv	5’ GTCTAAGCCAGGCACGTAAAGG 3’
GH3.5-q-fw	5’ GTGGTCACAACTTATGCAGGTCTC 3’
GH3.5-q-rv	5’ GCGCCTTGTTCTTGAACCCAAC 3’
YUC1-q-fw	5’CAAAACACCCTTTCCTAACG 3’
YUC1-q-rv	5’TGCCCTTGATTGTCTTCC 3’
YUC2-q-fw	5’CAACATTCGGAGTAGCCA 3’
YUC2-q-rv	5’GCACCAACATCCAACACT 3’
TAA1a-q-fw	5’CCCCTAACAATCCAGACG 3’
TAA1a-q-rv	5’GACACGGTGAAGAGCATAA 3’
PtrMIR319a-pro-fw	5’CCCTCCAAAGAACATATAACGTGTTTC 3’
PtrMIR319a-pro-rv	5’CTTCCAAACTCGGTTGTGACTTTGA 3’
